# Supplementary material for: LncRNA PART1 Promotes Proliferation and Migration, Is Associated with Cancer Stem Cells, and Alters the miRNA Landscape in Triple-Negative Breast Cancer
Source: Cancers (Basel). 2021 May 27;13(11):2644. doi: 10.3390/cancers13112644 (PMC8198907; doi:10.3390/cancers13112644)
Supplement: Supplementary file 1 [file cancers-13-02644-s001.zip › cancers-1244158-supplementary.pdf]

Supplementary Materials: LncRNA *PART1* Promotes Proliferation and Migration, is Associated with Cancer Stem Cells, and Alters the miRNA Landscape in Triple-Negative Breast Cancer

Brianne M. Cruickshank, Marie-Claire D. Wasson, Justin M. Brown, Wasundara Fernando, Jaganathan Venkatesh, Olivia L. Walker, Fiorella Morales-Quintanilla, Margaret L. Dahn, Dejan Vidovic, Cheryl A. Dean, Carter VanIderstine, Graham Dellaire and Paola Marcato

Table S1. Gene-specific primers.

Gene Name	Primer Sequence (5′ to 3′)	
	Forward	Reverse
PART1	CAGGGTACGCCAACTATAGGAC	TTCAGCTTTCCAGAGCCAGT
NEAT1	CCTCCCTTTAACTTATCCATTCAC	TCTCTTCCTCCACCATTACCA
DANCR	AGGAGTTCGTCTCTTACGTCT	TGAAATACCAGCAACAGGACA
ARF1	GTGTTCGCCAACAAGCAGG	CAGTTCCTGTGGCGTAGTGA
PUM1	GGCGTTAGCATGGTGGAGTA	CATCCCTTGGGCCAAATCCT
B2M	AGGCTATCCAGCGTACTCCA	CGGATGGATGAAACCCAGAC
GAPDH	GGAGTCAACGGATTTGGTCGTA	TTCTCCATGGTGGTGAAGA
ACSL3	TGTTGATGGAAAGCCACCGA	GTTTTCCATGCTGGCCTTGG
GPR89B	TGCTGTCAACTGCCCATACA	GCCGTTCCAGGGCTAGAATA
LOC101928401	GTACTCCACGGCTACATGCT	TCCACGGAGATTCTGCTCTTG
PPP2R3A	GTTGAGAACGATGGGCCTGA	TTGTGCTTGGGCAGATTCCT
BICCI	GGCCATGTTACAAGCTGCTG	TGGCCAAGCAATCTGCGTAT
CDKAL1	CCTCCATCAGCAAACCGCTA	CTGAGATGCAGCAGAGGTGT
KLK5	TTTTCAGAGTCCGTCTCGGC	GGATGGATTTGACCCCCTGG
MYO5A	GTGAGCGAGGAGCTTGATGT	TCATCCTTGGGTTGGATGGC
SUPT3H	GCCACATCAGAGAGGCCATT	TTCCTGCGGTAGGCATTTGT
ZHX2	ATGTCGTGTCCATCACCACC	CAGGCTTCATGATGGGGGTT

Table S2. Oligos for cloning into pmirGLO Dual-Luciferase miRNA Target Expression Vector.

Oligo Name	Sequence (5′ to 3′)
PART1-WT F	GCGGCCGCCTTCCCACAGTTTTCCAAAAATGACTCAC
PART1-WT R	TCGAGTGAGTCATTTTTGGAAAACTGTGGGAAGGCGGCCGCAGCT
PART-1 MUT F	GCGGCCGCCTTCCCACAGTTTTCCAAAAAGTCGGCAC
PART-1 MUT R	TCGAGTGCCGACTTTTTGGAAAACTGTGGGAAGGCGGCCGCAGCT

Table S3. Associations with *PART1* with hazard ratio (HR) in breast cancer patient datasets based on median expression and survival.

Subtype	HR	Lower CI	Upper CI	*p*-value	Sample #
**Cell 2015**					
Basal	2.82	0.88	9	0.08	107
Her2	1.15	0.45	2.9	0.77	120
LumA	0.57	0.26	1.3	0.17	200
LumB	0.84	0.31	2.3	0.74	122
TNBC	1.38	0.46	4.1	0.56	82
non-TNBC	0.95	0.65	1.4	0.8	734
All samples	1.09	0.76	1.6	0.64	816
**PanCancer Atlas**					
**Subtype**	**HR**	**Lower CI**	**Upper CI**	***p*-value**	**Sample #**
Basal	1.45	0.63	3.4	0.38	171
Her2	1.85	0.62	5.5	0.27	78
LumA	0.63	0.37	1.1	0.082	499
LumB	0.75	0.36	1.5	0.43	197
All samples	0.92	0.67	1.3	0.59	1084
**METABRIC**					
**Subtype**	**HR**	**Lower CI**	**Upper CI**	***p*-value**	**Sample #**
Basal	1.07	0.74	1.6	0.72	199
claudin-low	0.80	0.53	1.2	0.3	199
Her2	0.95	0.69	1.3	0.77	220
LumA	0.99	0.8	1.2	0.91	679
LumB	1.07	0.85	1.3	0.58	461
All samples	1.00	0.89	1.1	0.97	1758
**KMPLOT, probe 205833_s_at**					
**Subtype**	**HR**	**Lower CI**	**Upper CI**	***p*-value**	**Sample #**
Basal	1.49	1.15	1.92	0.0021	618
Her2	1.17	0.8	1.72	0.42	251
LumA	0.87	0.73	1.03	0.11	1933
LumB	0.95	0.79	1.15	0.61	1149
TNBC	1.38	0.9	2.12	0.13	255
All samples	0.87	0.73	1.03	0.11	3951

Breast cancer patient survival data based on high versus low *PART1* expression (stratified based on median expression) in tumors (RNA-seq for PanCancer Atlas and Cell 2015 dataset, and microarray for METABRIC and KMPlotter dataset) for all breast cancer patients in each cohort or in a subset of patients based on molecular subtype. For the KMPlotter analysis, two probes for *PART1* were available and assessed (205833_s_at, 205834_s_at). Hazard ratio (HR) was calculated based on the median cutoff between patient tumors with high versus low expression levels of *PART1*. The sample # represents the number of patient tumor samples. Confidence interval is abbreviated CI in the table.

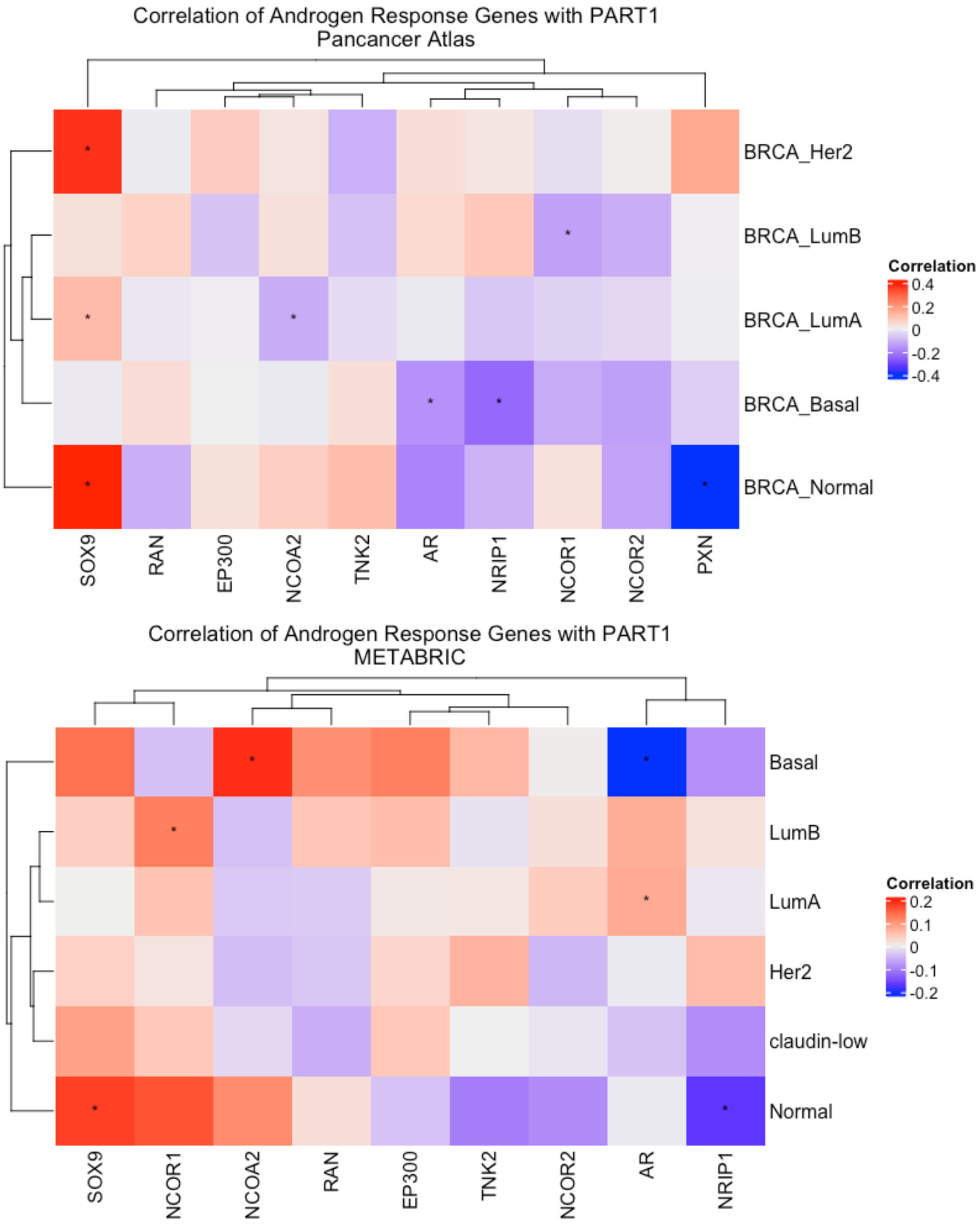

Figure S1. *PART1* is associated with androgen response genes. Spearman correlations of *PART1* expression with the expression of androgen response genes in breast cancer patient tumors in two datasets: (**A**) TCGA PanCancer Atlas and (**B**) METABRIC. The correlation was calculated with the cor.test() function in Rv4.2. * indicates a p value of < 0.05.

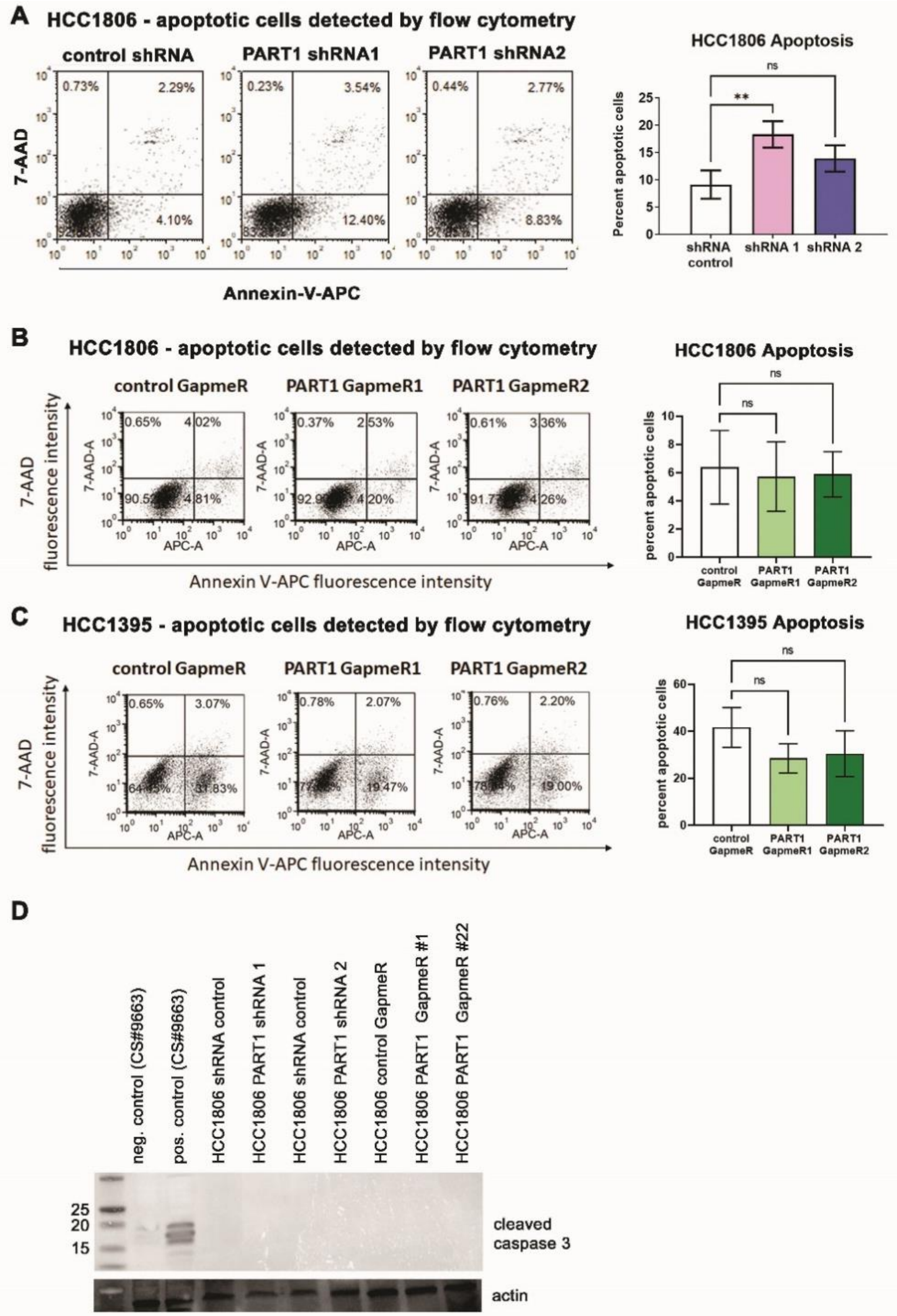

Figure S2. *PART1* knockdown induces minimal effects on apoptosis. (**A**, **B** and **C**) Representative flow cytometry dot plots of HCC1806 scramble control shRNA clone or HCC1806 *PART1* shRNA1 or 2 clones (**A**, $n = 3$), HCC1806 cells treated with negative control GapmeRs or *PART1* GapmeR#1 or #2 (**B**, $n = 3$), or HCC1395 cells treated with negative control GapmeRs or *PART1* GapmeR#1 or #2 (**C**, $n = 3$) stained with 7AAD and Annexin V-APC. The percentage of apoptotic cells (all annexin-V positive stained cells) was quantified (significance determined by one-way ANOVA, followed by a Dunnett's post-test for multiple comparisons). Error bars represent standard deviation. (**D**) Cleaved caspase 3 is detected via western blotting in control lysates but not in HCC1806 cells with *PART1* shRNAs or GapmeR treatment. ** indicates a p value of < 0.01, ns indicates a non-significant p value.

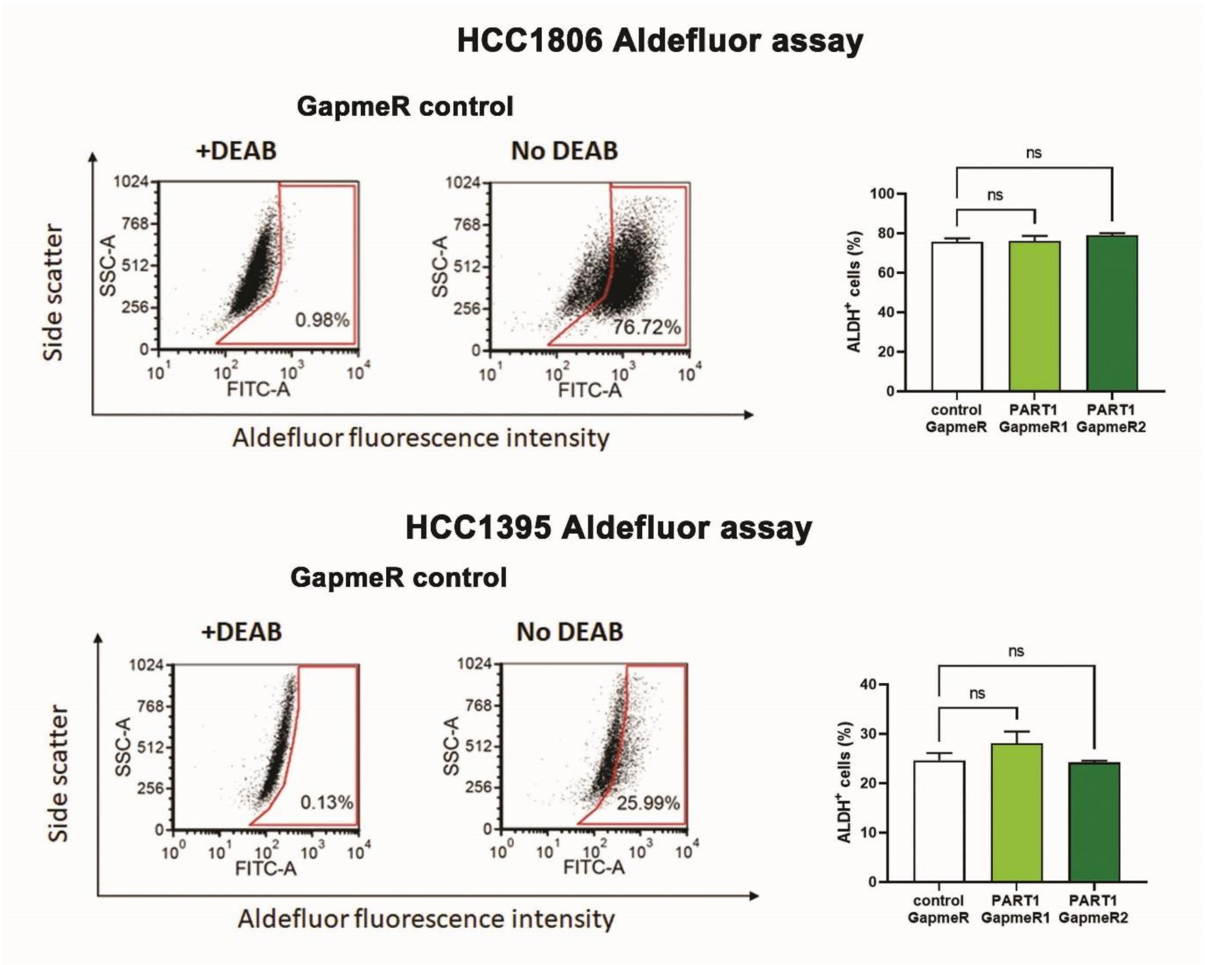

Figure S3. *PART1* GapmeR treatment does not change the percentage of Aldefluor^{high} cells in HCC1806 and HCC1395 cells. Representative flow cytometry dot plots of HCC1806 and HCC1395 cells treated with control GapmeRs for 48 h and then assessed for the percentage of Aldefluor^{high} cells. Debris and dead (positive 7AAD staining) were gated out of the analysis. The bar graphs show the average percentage of Aldefluor^{high} cells. Significance was determined by one-way ANOVA, followed by a Dunnett's post-test for multiple comparisons (ns indicates a non-significant *p* value).

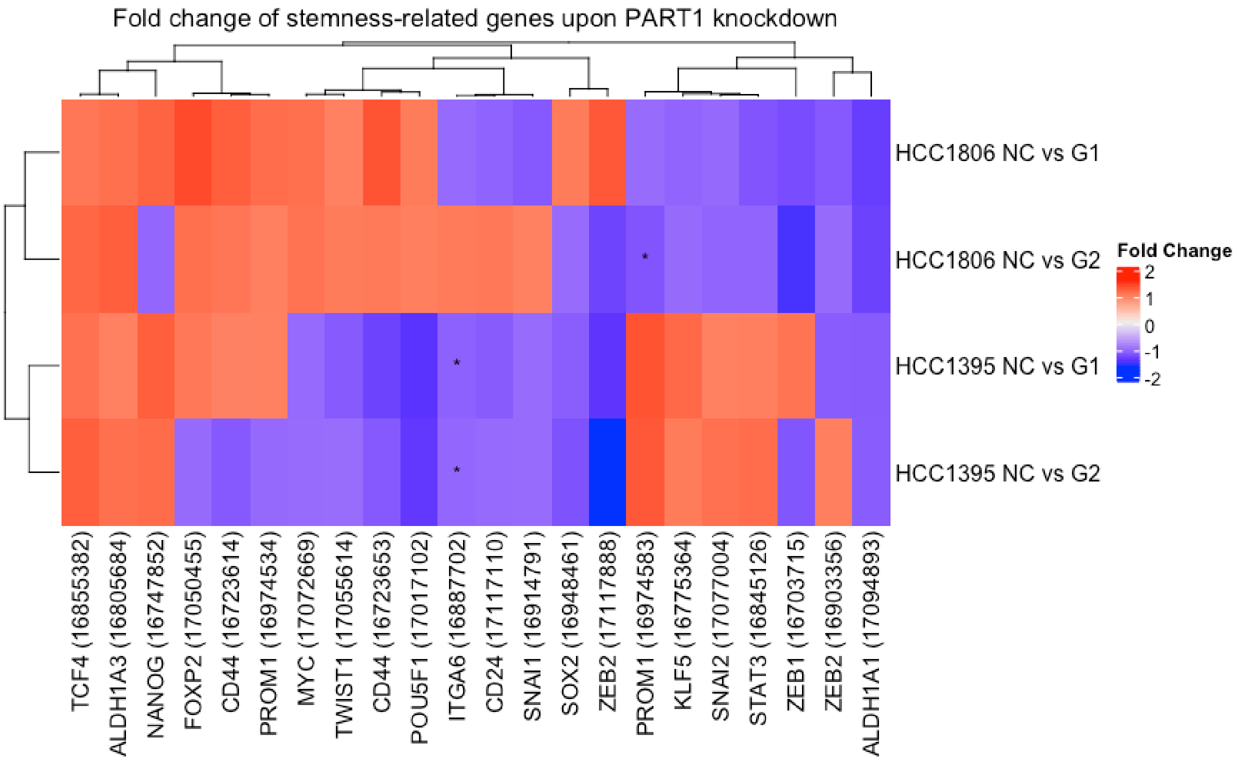

Figure S4. *PART1* knockdown has minimal effects on expression of CSC markers and stemness genes in HCC1806 and HCC1395 TNBC cells. Transcriptome changes induced by *PART1* knockdown (control (NC) versus GapmeR #1 (G1) and GapmeR #2 (G2)) were quantified in HCC1806 and HCC1395 cells using the Affymetrix Human Gene 2.0 ST microarray platform ($n = 3$). The Affymetrix probe ID is shown in parentheses next to the gene name to differentiate multiple probes to the same transcript. The heatmap shows the log base 2-fold change (log2FC) of stemness-related genes upon *PART1* knockdown. * indicates a fold change p value of < 0.05.

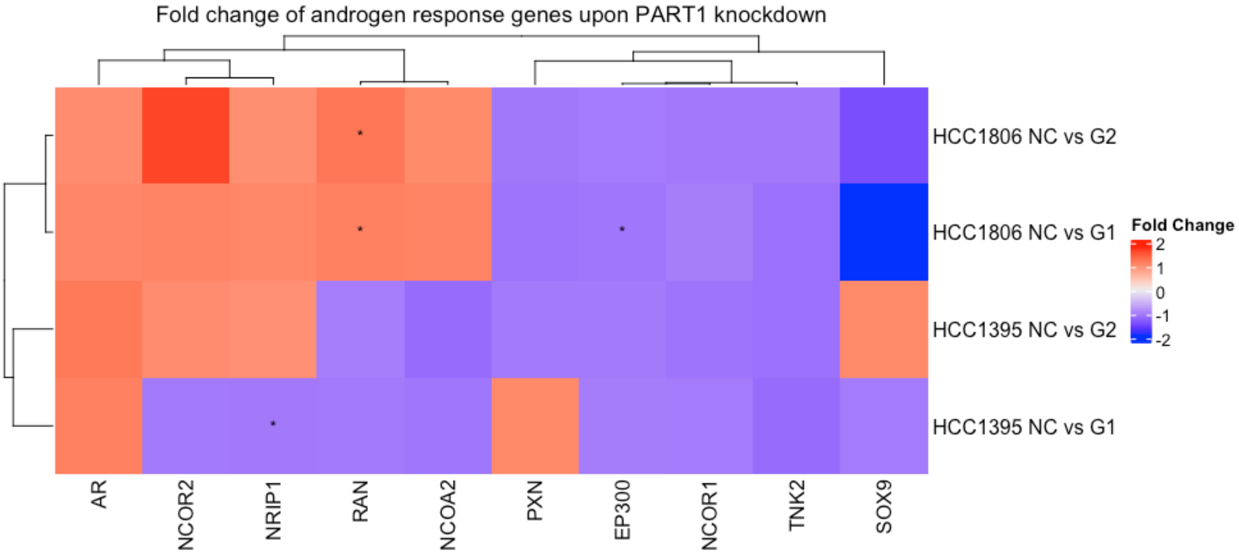

Figure S5. *PART1* knockdown induces minimal changes in gene expression of androgen signaling genes in HCC1806 and HCC1395 TNBC cells. Transcriptome changes induced by *PART1* knockdown (control (NC) versus GapmeR #1 (G1) and GapmeR #2 (G2)) were quantified in HCC1806 and HCC1395 cells using the Affymetrix Human Gene 2.0 ST microarray platform ($n = 3$, # represents the word number). The heatmap shows the log base 2-fold change (log2FC) of androgen response genes upon *PART1* knockdown. * indicates a fold change p value of < 0.05.

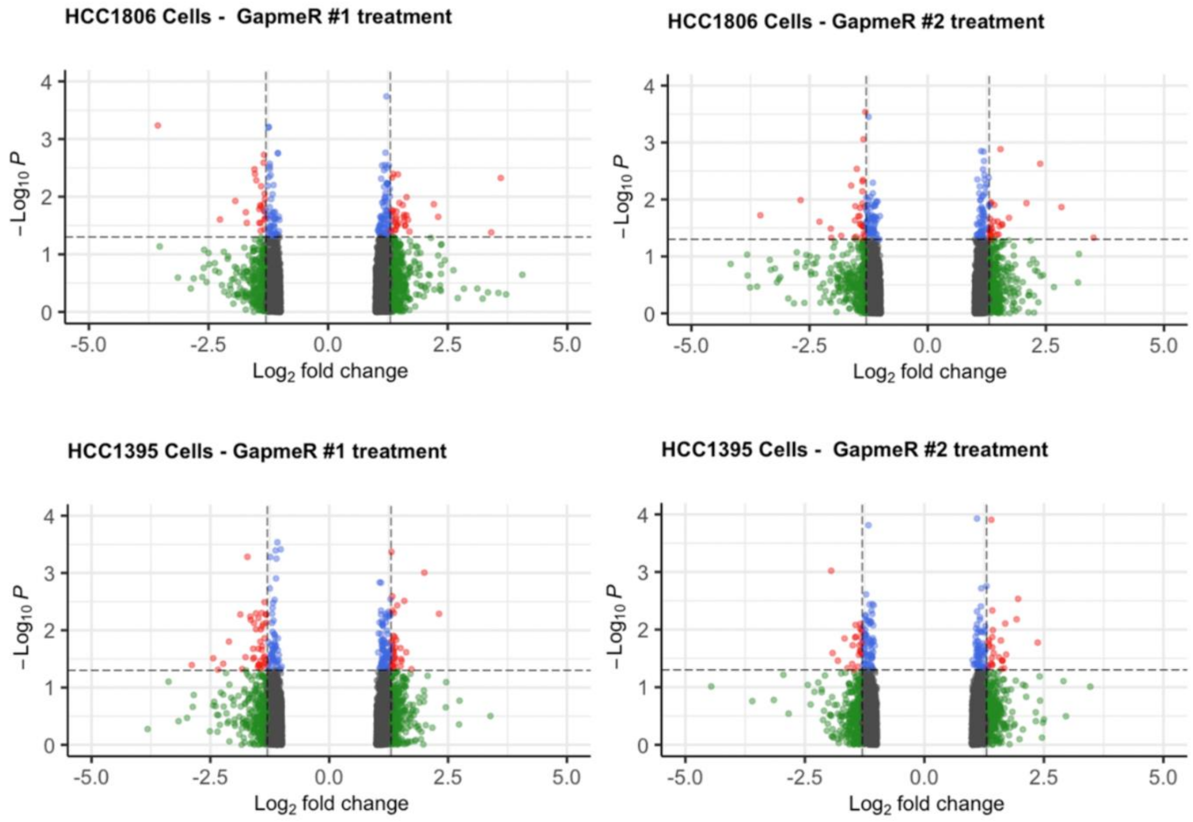

Figure S6. Volcano plots of miRNA fold change and *p* value from 4.0 miRNA gene chip array. The fold change thresholds are set at +/−1.3 and the *p* value threshold is set at 0.05. The # symbol specifies the GapmeR treatment used to knockdown *PART1* (where GapmeR #1 refers to an oligonucleotide with the following sequence: ATTCCAGATAAGTAGA; and GapmeR #2 refers to an oligonucleotide with the following sequence: GTGATTCCAGAATAAGT).

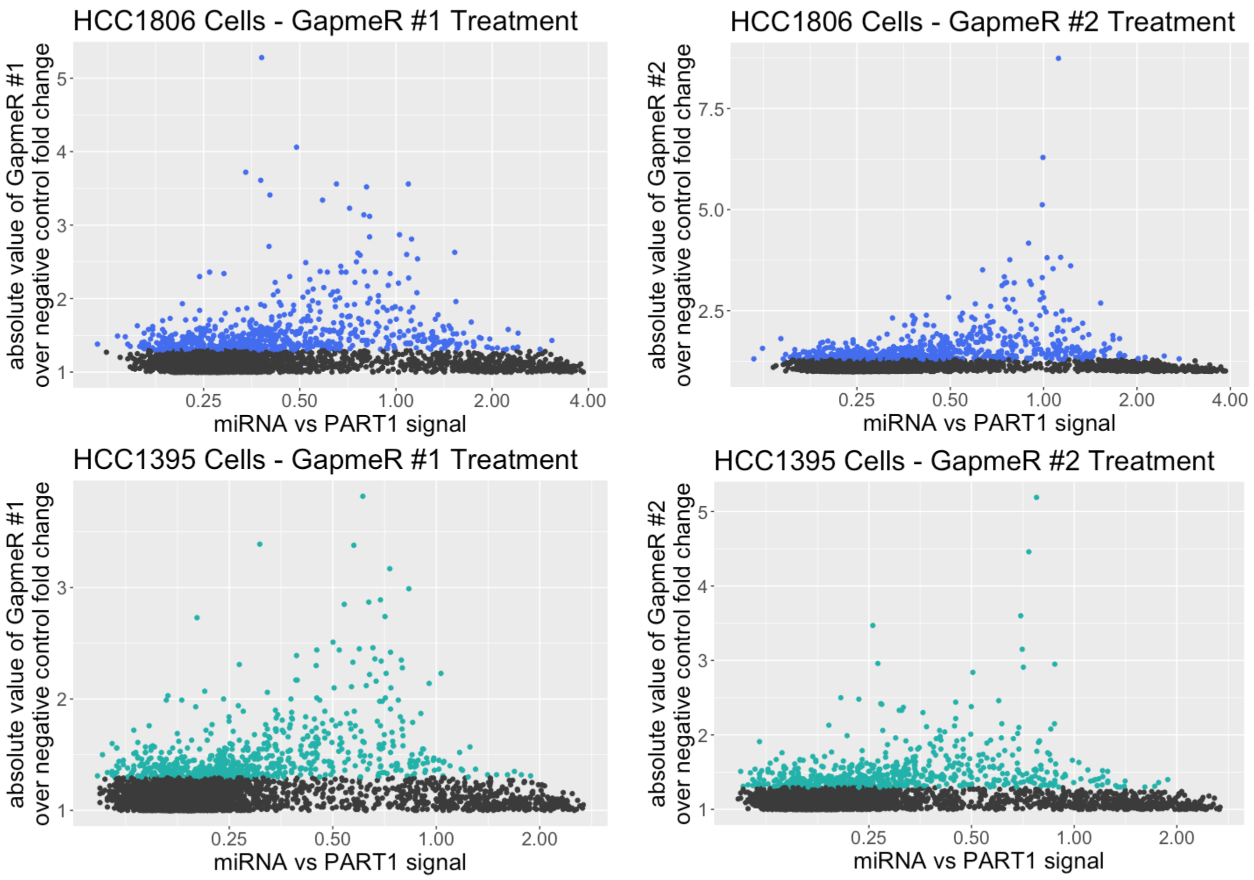

Figure S7. miRNA versus *PART1* abundance. The relative abundance of all the miRNAs in the 4.0 miRNA gene chip array to *PART1* (abundance extrapolated from GSE156114 and GSE163569) detected in the gene chips in the negative control samples was calculated for HCC1806 and HCC1395 cells (average of 3n). The # symbol specifies the GapmeR treatment used to knockdown *PART1* (where GapmeR #1 refers to an oligonucleotide with the following sequence: ATTCCAGA-TAAGTAGA; and GapmeR #2 refers to an oligonucleotide with the following sequence: GTGATTCCAGAATAAGT).

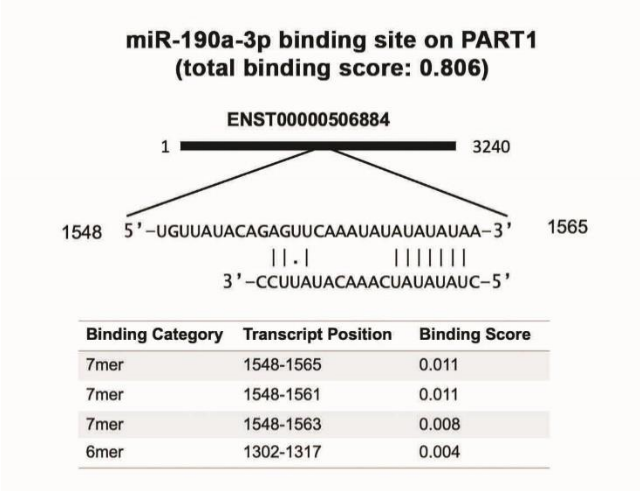

Binding Category	Transcript Position	Binding Score
7mer	1548-1565	0.011
7mer	1548-1561	0.011
7mer	1548-1563	0.008
6mer	1302-1317	0.004

Figure S8. The LncBase v2 predicted *PART1* binding sites and affinity threshold score of *miR-190a-3p*.

Files S1–5. are provided separately attached as Excel Files.
